# Abdominal vascular emergencies: US and CT assessment

**DOI:** 10.1186/2036-7902-5-S1-S10

**Published:** 2013-07-15

**Authors:** Eugenio Annibale Genovese, Paolo Fonio, Chiara Floridi, Monica Macchi, Anna Maccaferri, Antonio Amato Stabile Ianora, Lucio Cagini, Gianpaolo Carrafiello

**Affiliations:** 1 Department of Radiology, University of Cagliari, Cagliari, Italy; 2 Institute of Diagnostic and Interventional Radiology, University of Turin, Turin, Italy; 3 Department of Radiology, University of Insubria, Varese, Italy; 4 Diagnostic Imaging Section, University of Bari, Bari, Italy; 5 Thoracic Surgery Unit, University of Perugia, Perugia, Italy

## Abstract

Acute vascular emergencies can arise from direct traumatic injury to the vessel or be spontaneous (non-traumatic).

The vascular injuries can also be divided into two categories: arteial injury and venous injury.

Most of them are life-treatening emergencies, sice they may cause an important ipovolemic shock or severe ischemia in their end organ and require prompt diagnosis and treatment.

In the different clinical scenarios, the correct diagnostic approach to vascular injuries isn’t firmly established and advantages of one imaging technique over the other are not obvious.

Ultrasound (US) is an easy accessible, safe and non-invasive diagnostic modality but Computed Tomography (CT) with multiphasic imaging study is an accurate modality to evaluate the abdominal vascular injuries therefore can be considered the primary imaging modality in vascular emergencies.

The aim of this review article is to illustrate the different imaging options for the diagnosis of abdominal vascular emergencies, including traumatic and non traumatic vessel injuries, focusing of US and CT modalities.

## Introduction

Acute vascular emergencies can arise from direct traumatic injury to the vessel or be spontaneous (non-traumatic).

The vascular injuries can also be divided into two categories: arteial injury and venous injury.

The findings of various types of vessel injury include laceration with active hemorrage, occlusion, and, for arteries formation of psedoaneurysm and dissection.

Most of them are life-treatening emergencies, sice they may cause an important ipovolemic shock or severe ischemia in their end organ and require prompt diagnosis and treatment.

In the different clinical scenarios, the correct diagnostic approach to vascular injuries isn’t firmly established and advantages of one imaging technique over the other are not obvious.

Ultrasound (US) is an easy accessible, safe and non-invasive diagnostic modality and is presently cosidered with the use of Doppler duplex sonography, the first-line examination for evaluation of vascular injuries [1].

Computed Tomography (CT) with multiphasic imaging study is an accurate modality to evaluate the abdominal vascular injuries, quick to perform, non-invasive, readily avaible and able to visualize other anatomical regions simultaneously [2]. Certainly CT can be considered the primary imaging modality in politraumatic patient with suspicious vascular trauma [3].

The aim of this review article is to illustrate the different imaging options for the diagnosis of abdominal vascular emergencies, including traumatic and non traumatic vessel injuries, focusing of US and CT modalities.

## Traumatic arterial emergencies

### Traumatic aortic injuries

Traumatic aortic injury following blunt (nonpenetrating) trauma is a rare but potentially lethal injury. Recent autopsy series have shown the incidence of abdominal aortic injuries ranging from 12 to 15%[4]; abdominal trauma represents only 4–6% of aortic injuries, the remainder involving the thoracic aorta [5]. The retroperitoneal position of the abdominal aorta explains the rarity of this event; because of this location, the abdominal aorta is indeed protected anteriorly by the abdominal wall and the visceral organs and posteriorly and laterally by the vertebrae and the thick paravertebral musculature [6].

Intimal disruption is the most common type of blunt aortic damage observed, with the distal intimal flap often dissected by the blood flow, leading to thrombosis [7].

Occasionally, frank transection and disruption of the aorta with exsanguinating hemorrhage may happen [5,8,9].

Injuries are typically limited in length. The anatomic location of abdominal aortic injury is usually infrarenal because the suprarenal abdominal aorta is better protected by the lower bony thorax. The most frequent sites of injury are at the level of the inferior mesenteric artery (33%), near the renal arteries (24%), and between the inferior mesenteric artery and bifurcation (19%) [10].

A high index of clinical suspicion is therefore essential because rapid diagnosis and treatment are crucial for a successful outcome in a patient with arterial injury that is potentially life- or limb-threatening. The triad of blunt abdominal trauma, acute lower extremity arterial insufficiency, and lower extremity paralysis could suggest the diagnosis [11].

Among hemodynamically unstable patients, few investigations can actually be instituted. Ultrasonography has been proposed as a useful screening tool for the detection of intraabdominal fluid [12]; however, very little detailed information can be obtained from retroperitoneum and major abdominal vessels [13].

Hemodynamically stable patients and patients who respond to initial resuscitation measures require instead further diagnostic evaluation. MDCT has recently become the modality of choice for a comprehensive evaluation of patients with acute vascular disease after blunt trauma, completely replacing in the past years angiography, traditionally considered the gold standard, as a diagnostic tool [14].

Compared to conventional angiography, MDCT indeed has many advantages; it is fast, minimally invasive, and readily available in most large trauma centers. In addition, it is capable of documenting associated abdominal injuries and extrinsic lesions impacting the vasculature can be seen directly. There is still the disadvantage of the need for intravenous contrast, but on the other hand, it can be used as the final diagnostic test on which definite treatment, whether open surgery or endo-vascular stent grafting, in cases of hemodynamically stable patients with intimal injuries or dissections and preserved flow through the vessel, can be planned [11,15].

Direct signs of abdominal aortic injury include the presence of a large intraluminal flap, intramural hematoma, focal intimal injury, pseudoaneurysm formation, or active extravasation of contrast material; indirect findings of abdominal aortic injury include the presence of retroperitoneal hematoma as well as surrounding retroperitoneal or mesenteric stranding [16].

The finding of frank rupture of the aorta with exsanguinating hemorrhage is extremely uncommon among those patients reaching the hospital alive, and clearly represents an indication to urgent surgical repair.

Given the frequent association of abdominal aortic injury and other solid organ and osseous injuries, it is particularly important to include the abdominal aorta in a CT search pattern in patients with severe traumatic injuries. Close inspection of the aorta is particularly important in patients with atherosclerosis, hypertension, or Marfan’s disease, as the intima tears more easily in patients with these conditions [17,18].

### Visceral arteries trauma

Most renal artery pseudoaneurysms or dissections result from penetrating injuries, many of which are iatrogenic (i.e., associated with renal biopsy or nephrostomy tube placement) [19]. Hypertension and rupture with hemorrhage are the most important complications. Delayed hemorrhage (days to weeks after the initial injury) is not rare and may be heralded by hematuria.

**Figure 1:**
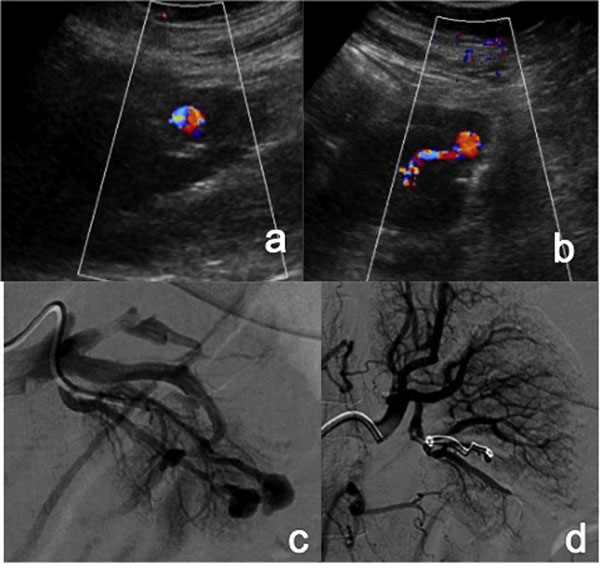
Renal Iatrogenic Pseudoaneurysm (a-b) Color-Doppler sonography images show an altered flow in a branch of left renal artery in a patient undergone to percutaneous renal biopsy. (c) Selective left renal arteriography shows two distal renal pseudoaneurysms. (d) the angiogram performed at the end of the embolization procedure (carried out with microcoils) revealed complete exclusion of the PSAs.

Color-flow and gray-scale Doppler sonography can be used to diagnose and follow renal artery pseudoaneurysm; pseudoaneurysm appears as a rounded anechoic structure on gray-scale images, with to-and-fro swirling on color-flow images. [Figure 1]

CT is highly reliable for diagnosing renal parenchymal and pyelocalyceal injuries, main renal arterial occlusion, and active bleeding. CT is not as accurate for diagnosing branch arterial injuries, including pseudoaneurysm or arteriovenous fistula. In kidneys that develop pseudoaneurysms, the initial CT scan often shows parenchymal laceration without pseudoaneurysm, because acute thrombus may temporarily seal the laceration. Over several days or weeks, clot lysis occurs with subsequent formation of a pseudoaneurysm. A typical pseudoaneurysm enhances in the arterial phase and washes out in the delayed phase**;** pseudoaneurysms are seen as focal, rounded regions equal in attenuation to the vessel or surrounding arterial structures on an arterial phase image [20]. Multiphasic acquisitions are helpful for differentiating active extravasation from pseudoaneurysms. On delayed phases, foci of extravasated blood are typically larger, and the relative hyperattenuation persists throughout the various phases of image acquisition, whereas pseudoaneurysms are identical in size and shape and the attenuation is similar to the aorta in all phases, washing out on later phases of image acquisition.

This differentiation has important therapeutic implications: active bleeding requires urgent endovascular or surgical management whereas pseudoaneurysms may be treated in a semiurgent manner [21].

Pseudoaneurysm of the lumbar artery is rare in the medical literature and is usually described after penetrating injuries. It is also reported following blunt trauma, percutaneous renal interventions, laparoscopic splenectomy, or spontaneously [22,23].

In most reported cases, lumbar artery injury was not detected during first examination and was diagnosed because of symptoms caused by the mass effect of pseudoaneurysm. Doppler US is useful in depicting typical flow pattern inside the aneurysm, whereas CT angiography is helpful in determining the origin and dimensions of an aneurysm and planning the therapeutic approach.

Pseudoaneurysm of the lumbar artery can present as increased density or as soft tissue thickening in the psoas muscle region on unenhanced CT scans. The lumen of the aneurysm can be demonstrated on CT scan following bolus contrast injection. MDCT angiography is very helpful in depicting the extension of the aneurysm, its relation to the feeding vessel, and in planning the treatment; CT angiography also showed the level and diameter of the feeding artery of pseudoaneurysm [24].

### Vascular pelvic trauma

Patients who suffer major blunt pelvic trauma and sustain displaced fractures have a high risk of major pelvic vascular injuries, with significant mortality and morbidity [25]. Approximately 40% of patients with a pelvic fracture may have an associated pelvic vascular injury and hemorrhage is the leading cause of mortality in 60% of cases [26]. The rupture of iliac arteries is an uncommon but life-threatening consequence of pelvic trauma [27]. The incidence of iliac artery injury has been reported as 0.4% of the total arterial trauma [28]. In a study involving 657 iliac interventions from 1981 to 2000, Allaire et al. reported that the incidence of rupture was 0.8% [29]. Rupture may be an acute event with signs of internal hemorrhage (groin and back pain and hemodynamic changes) or may arise secondarily and form a false aneurysm [30].

Rapid detection and assessment of pelvic vascular injury afforded by the shorter acquisition times and increased spatial resolution of MDCT are useful for properly triaging critically injured trauma patients, allowing also an overall evaluation of multiple parenchymal and orthopedic lesions [31]. However, in patients with poor hemodynamic conditions and iliac artery injury is highly suspected, US should be preferred [32].

**Figure 2:**
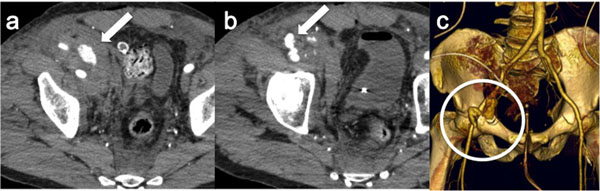
Traumatic rupture of the external iliac artery (a-b) CT-angiography images show the rupture of the right external iliac artery with contrast medium extravasation into the iliac muscle. (c) Volume Rendering reconstruction (anterior-posterior view) from the same CT study confirms the contrast medium exstravasation (white circle).

Pelvic multiphasic CT allows for accurate differentiation between arterial and venous injury. On a portal venous phase image, an arterial hemorrhage [Figure 2] should have a higher attenuation than from a venous source, but significant overlap makes this distinction difficult Contrast extravasation seen on a portal venous phase image is defined as an arterial hemorrhage if it is present on the earlier, arterial phase image. Contrast extravasation seen on a portal venous phase image but not on the earlier arterial phase image is more likely venous in nature [33].

## Non traumatic arterial emergencies

### Rupture and impending rupture of abdominal aortic aneurysms

Aneurysmal dilatation of the abdominal aorta is a disease of aging and is rare before age 50 but is found in 2%–4% of the population older than 50 years [34]. The average age at the time of diagnosis is 65–70 years, and more men than women are affected. Concurrent coronary artery disease and peripheral vascular disease, as well as a family history of abdominal aortic aneurysm, are strong risk factors for the development of this condition.

A true aortic aneurysm is a localized dilatation of the aorta caused by weakening of its wall; it involves all three layers (intima, media, and adventitia) of the arterial wall. A pseudoaneurysm (false aneurysm) is a collection of flowing blood that communicates with the arterial lumen but is not enclosed by the normal vessel wall; it is contained only by the adventitia or surrounding soft tissue. Aneurysms may develop in any segment of the aorta, but most involve the aortic segment below the renal arteries. An aortic diameter of 3 cm or more is used to define an abdominal aortic aneurysm [35].

Prompt diagnosis of rupture and impending rupture of abdominal aortic aneurysms is imperative.

Patients who present with abdominal pain, a large abdominal aortic aneurysm, and no frank rupture pose a diagnostic dilemma. The symptoms may be attributable to aneurysmal instability, impending rupture, or a contained leak.

US plays a limited role in the assessment of acute aortic abnormalities.

Frequently, the entire aorta cannot be evaluated because of overlying bowel gas and body habitus. In addition, US is operator dependent, and the necessary expertise may not be readily available. A bedside examination with US may be helpful for patients whose condition is too unstable to allow their transfer to the CT scanner. US may help determine the size of the aneurysm and help identify hemoperitoneum. However, the utility of US for identifying an impending rupture or a contained rupture of an aneurysm is limited.

CT is the modality of choice for evaluation of acute aortic syndrome, because of the speed of the examination and the widespread availability of CT.

Unenhanced CT may help detect an aneurysm rupture by depicting an abdominal aortic aneurysm with surrounding retroperitoneal hemorrhage.

Contrast-enhanced CT provides additional information about the size of the aneurysmal lumen, presence of active extravasation, and relationship of the aneurysm to the celiac, superior mesenteric, renal, and inferior mesenteric arteries. A retroperitoneal hematoma adjacent to an abdominal aortic aneurysm is the most common imaging finding of abdominal aortic aneurysm rupture [36].

Periaortic blood may extend into the perirenal space, pararenal space, or the psoas muscles. Intraperitoneal extension may be an immediate or a delayed finding. These findings are readily visible on unenhanced CT images, which may have been obtained for another indication or as part of an aneurysm evaluation protocol. On contrast-enhanced CT images, active extravasation of contrast material is frequently demonstrated. An important imaging feature that may be seen in a contained rupture of an abdominal aortic aneurysm is the draped aorta sign [37].

This sign is considered present when the posterior wall of the aorta either is not identifiable as distinct from adjacent structures or when it closely follows the contour of adjacent vertebral bodies.

**Figure 3:**
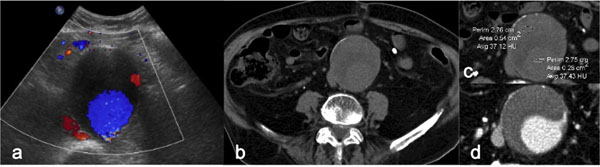
Impending aortic aneurysm rupture (a) Color-Doppler-sonography shows a large aortic aneurysm with intra thrombus flow (b) Axial unenhanced CT image depicts an abdominal aortic aneurysm with a hyperattenuating crescent sign (arrow), (c) and axial contrast-enhanced (b) which repre- sents an acute hematoma within the aneurysm wall

Findings Predictive of Impending Rupture The most common finding predictive of rupture is the maximum diameter of the aneurysm [38]. A patient with a very large abdominal aortic aneurysm (diameter of 7 cm) who presents with symptoms of acute aortic syndrome has a high likelihood of aneurysm rupture. Nonruptured aneurysms generally contain more thrombus than do ruptured aneurysms, and the thrombus-to-lumen ratio decreases with increasing aneurysm size [39]. A focal discontinuity in circumferential wall calcifications is more commonly observed in unstable or ruptured aneurysms [36]. A well-defined peripheral crescent of increased attenuation within the thrombus of a large abdominal aortic aneurysm is a CT sign of acute or impending rupture [Figure 3] [40]. This finding is best appreciated on unenhanced CT images. It represents an internal dissection of blood into either the peripheral thrombus or the aneurysm wall, a process that either causes or results from a loss in the ability of the thrombus to protect the aneurysm from rupture. It is one of the earliest and most specific imaging manifestations of the rupture process [36,41].

### Acute aortic dissection

Acute or type B aortic dissection is a life-threatening condition and must be diagnosed and treated promptly.

An aortic dissection arises from a tear in the intima which results in a separation of the aortic wall layers with infiltration of bleeding and the danger of aortic rupture. Various genetic disorders of connective tissue promote degeneration of the aortic media, most notably Marfan syndrome. Risk factors for aortic dissection are nicotine abuse, arterial hypertension, age and male gender. The clinical course and symptoms of aortic dissection are very dependent on the section of the aorta affected and the manifestations are manifold. Acute aortic dissection is in 80 % of cases first manifested as sudden extremely severe pain. The diagnostics and subsequent course control can be achieved by a variety of imaging procedures but the modality of choice is CT [42]. Findings of a contrast-enhanced double lumen and an intimal flap in the aorta are diagnostic. In dynamic occlusion, the intimal flap prolapses across the branch-vessel origin and covers the lumen like a curtain. Reliable identification of true lumen and false lumen are important for treatment planning. Le Page et al [43] described the beak sign—a triangular area of high attenuation—and a large crosssectional area on contrast enhanced CT images as the most useful indicators of the false lumen in acute and chronic aortic dissection. Less common and less reliable identifiers of the true and false lumina are patterns of eccentric calcification (ie, calcification in the dissection membrane facing only one lumen), intraluminal thrombus, and the cobweb sign (ie, thin linear intraluminal filling defects).

### Aortoenteric fistulas

Aortoenteric fistula is a rare but potentially fatal entity, presents a significant challenge to radiologists in diagnosis, largely because of its subtle and nonspecific imaging findings. Primary aortoenteric fistulas are a complication of atherosclerotic aortic aneurysms, whereas secondary aortoenteric fistulas are a complication of aortic reconstructive surgery [44]; the secondary form is more common.

Most fistulas involve the duodenum, most commonly its third and fourth portions. Symptoms include abdominal pain, hematemesis, and melena.

**Figure 4:**
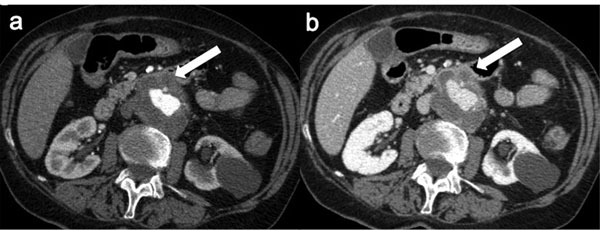
Aortoduodenal fistula (a) Arterial CT axial images shows the adhesion of the aortic wall with the duodenum. The aorta appears aneurysmal and its wall is irregular (arrrow). (b) in venous phase CT image the aortic wall presents marked contrast enhancement (arrow).

Primary aortoenteric fistulas may pose a diagnostic dilemma for the clinician, especially in the absence of gastrointestinal tract bleeding. Uppergastrointestinal- tract endoscopy may help rule out other causes of bleeding but rarely helps diagnose a fistula. Typical CT findings [Figure 4], which can overlap with those seen in perigraft infection, aortitis, infected/mycotic aneurysms, perianeurysmal fibrosis, and the immediate post-operative period after placement of a graft, include: effacement of the fat planes around the aorta, perigraft fluid/soft tissue thickening, ectopic gas, tethering of adjacent thickened bowel loops towards the aortic graft, and in rare cases, extravasation of contrast from the aorta into the involved segment of bowel [45].

### Spontaneous abdominal bleeding

Patients with lower INR are out of therapeutic effect being at risk of thrombosis, whilst an INR >5 increases the risk of bleeding more than 30% per year [46]. Due to the dramatic improvement of patients receiving anticoagulant therapy, it is important focussing on how to deal with possible risks of those therapies and mainly with the risk of spontaneous bleedings. When oral anticoagulant therapy is considered, some authors reported a risk major hemorrhagic event ranging from 0.2%-3% years per patient [47,48].

It may occur spontaneously or result from minor trauma and may affect any organ system.

Given the technical difficulty to identify by US the bleeding site and selectively bind the target vessel, an accurate iter based on a multiphasic CT examination has been widely accepted as an optimal strategy.

The bleeding site was defined as a non homogeneous density area (hematoma) with an attenuation value of 50-80 HU in non-contrast scan, whilst active constrast medium extravasation was defined as high-attenuation shapes on arterial phase that initially appeared like a jet or fountain with a tapered edge and after was confirmed on venous and late phases, where the contrast-enhanced blood was mixed with the fresh and clotted blood already present within the hematoma.

Renal hemorrhage may occur in suburothelial, intraparenchymal, subcapsular, or perinephric locations, or in a combination of these [49]. Acute hemorrhage is best depicted with nonenhanced CT.

However, an additional examination with contrast- enhanced CT should always be performed to exclude other pathologic renal conditions that may cause hemorrhage and may require specific therapy. The presence of perinephric hemorrhage is confirmed by bridging septa of the perinephric fat, which determine the location of bleeding.

Renal angiomyolipoma (AML) is one of the most frequent causes of subcapsular and perinephric heamorrhage in adults. AML is a relatively rare benign tumor (0.3-3% of all renal neoplasms) composed of fat, muscle tissue and vascular elements in various proportions [50].

The small-sized lesions are usually asymptomatic and are often an incidental diagnostic finding. Larger lesions may be clinically because, in 40% of cases, they tend to rupture with subsequent bleeding [51].

**Figure 5:**
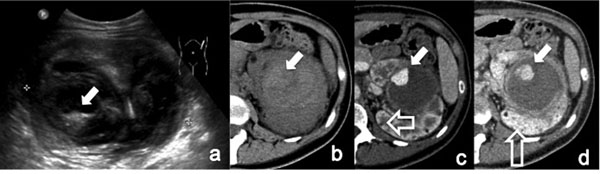
Angiomyolipoma bleeding (a) US image shows a large heterogeneous lesion with mixed areas hyper and hypo-echoic (b) unenhanced axial CT image confirm a large hyperdense lesion at upper left renal pole. (c-d) Arterial and venous phase CT axial images show contrast material extravasation (arrowheads) from the upper left renal pole into a large hematoma. The renal parenchyma close to the hematoma presents multiple lesions having attenuation characteristic of angiomyolipomas (hollow arrow) (patient with Tubrous Sclerosis).

Therefore a correct diagnostic approach is important in order to guide the surgical approach. Emergency US is often the first diagnostic test to be carried out and enables to assume the presence of a neoplasm with a reported sensitivity of 13-30% while identifying both the haemorrhage and the tumor, as compared to a reported sensitivity of 71% for the CT scan [50,51]. In cases of bleeding, at ultrasound AML appear as inhomogeneous, mainly iso- or hypoechoic perinephric masses inside whicha few hyperechoic areas may also be seen [Figure 5]. The CT aspect of these lesion is that of a perinephiric mass with inhomogeneous density, mainly hyperdense due to haemorrhage, with hypodense areas which are more or less outlined based on the fat content of the lesion [52]. In cases of profuse bleeding the hypodense areas may be masked by the hyperdensity of the haemorrhage; in this case thin scans (1-3 mm) of the lesion are crucial for detecting even minimum quantities of fat tissue [53].

### Acute mesenteric ischemia

Acute Mesenteric Ischemia (AMI) is a vascular emergency with comparable urgency to myocardial infarction or apoplexy.

AMI is the cause of acute abdomen in up to 10% of patients aged over 70. The following are predisposing risk factors: heart failure, atrial fibrillation, coronary heart disease, arterial hypertension, and peripheral arterial occlusion [54]. One of the most common causes of AMI is embolic arterial occlusion, implicated in up to 50% of cases, typically involving the superior mesenteric artery ( SMA ) just distal to the middle colic artery origin. This causes vasoconstriction of the arterial branches originating beyond the embolic occlusion, or additional distal embolization, further worsening the extent of bowel ischemia. The embolus usually is from a cardiac source and therefore risk factors such as myocardial infarction, arrhythmia, valvular disease and ventricular aneurysm should be considered and appropriately excluded [55]. Mesenteric ischemia that results from SMA thrombosis usually occurs when a preexisting atherosclerotic stenosis, typically at the vessel origin, reaches a critical diameter and acutely thromboses. The onset of symptoms is typically acute and consists of severe abdominal pain that is disproportionate to the findings on physical examination [56].

The mortality rate of acute mesenteric ischemia (AMI) is 50% to 70% and has remained at this high level for decades [57]. The reasons for this are on the one hand insufficient understanding of its clinical picture in differential diagnosis of abdominal pain, when it is not considered, and on the other hand an unacceptable time delay before treatment even when a diagnosis of AMI is considered [58].

This is often caused by the time-consuming use of inappropriate diagnostic procedures.

**Figure 6:**
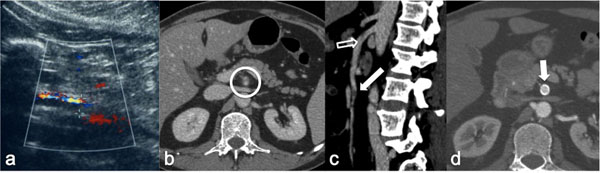
Spontaneous SMA dissection (a) Doppler sonography shows altered flow in SMA (sagittal view) (b)Contrast-enhanced CT axial scan shows an intimal flap in the SMA lumen (white circle). (c) Sagittal Reconstructed MPR from the same CT study confirms SMA intimal flap (hollow arrow) and shows also a distal thrombosis (white arrow). (d) Arterial phase CT axial image, obtained one month after stent implantation shows patent SMA stent (arrow) with obliteration of false lumen.

In acute mesenteric ischemia, due to distension of the intestinal loops, US should not be used for examination. However, Color Doppler US of mesenteric circulation has become a method which can be carried out in most patients. An experienced US operator can identify the celiac tripod and the superior mesenteric artery in 80-90% of patients [59] [Figure 6].

When acute occlusive mesenteric ischemia is suspected, biphasic contrast-enhanced CT with three dimensional multiplanar reconstruction ( MPR-CT) is the diagnostic tool of choice. CTA is more specific and will often demonstrate the site of embolic occlusion as a central filling defect within the SMA or may show an abrupt point where the artery is not opacified, with poor or absent collaterals [Figure 6].

Similar findings of arterial nonopacification are usually present in cases of SMA thrombosis, and there may be accompanying vascular calcifications at the origins of the celiac, SMA, and/or IMA along with well-formed collaterals [56].

CT may demonstrate bowel dilatation and wall thickening, as well as ascites and mesenteric edema in the early stages of AMI, whereas pneumatosis, pneumoperitoneum, and intravascular gas are seen in later stages. In cases of embolic occlusion, other abdominal organs such as the kidneys may show evidence of infarction.

### Rupture of visceral aneurysms

Visceral artery aneurysms (VAAs) are the intra abdominal aneurysms that affect the celiac artery, the superior and inferior mesenteric arteries, and the renal arteries and their branches.

**Figure 7:**
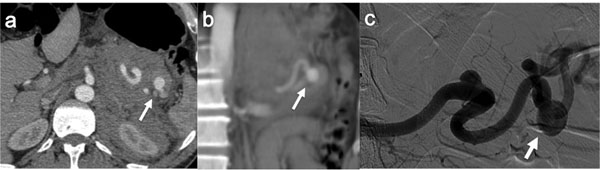
Spontaneous rupture of splenic aneurysm (a) Contrast enhanced axial CT image show a spontaneous rupture of splenic artery aneurysm with active contrast medium extravasation around the aneurysm. (b) Coronal MIP reconstruction of the same CT study. (c) Selective splenic arteriography confirms a large splenic aneurysm without active contrast medium extravasation (self limiting bleeding).

VAAs involve the splenic artery in 60%–80% of cases [Figure 7], the hepatic artery in 20%, the superior mesenteric artery (SMA) in 5.5%, the celiac artery in 4%, the gastric and gastroepiploic artery in 4%, the gastroduodenal artery and pancreatic branches in 6%, the jejunal and ileocolic arteries in 3%, and the inferior mesenteric artery in less than 1% [60]

True renal artery aneurysms (RAAs) are rare and traditionally have not been included in reviews of VAA because they have different clinical manifestations and are frequently associated with hypertension.

The advent of interventional procedures in the liver has made traumatic intrahepatic aneurysm the most frequently encountered VAA at some institutions [61].

The study by Stanley et al [62] reported rupture in up to 22% of cases, whereas recent reports include large numbers of asymptomatic patients in whom aneurysms are discovered incidentally [63].

The routine use of CT and US has led to the increased diagnosis of both symptomatic and asymptomatic VAAs. CT is powerful tools for diagnosis and treatment planning in patients with ruptured VAA. In fact, Increasingly faster speed and higher spatial resolution make CT a first-line imaging modality for patients who present to the emergency department with a suspicion of visceral aneurysms rupture. When a VAA ruptures, intraperitoneal or retroperitoneal hemorrhage or hematoma in a visceral organ or in the course of a visceral artery can be seen at CT. Although to our knowledge no reliable sign to suggest an impending rupture has been reported, rapid size increase in a known VAA may be predictive [64].

### Rupture of iliac artery aneurysm

Iliac artery aneurysms are rare, accounting for less than 2% of abdominal aneurysms. Common iliac artery is most commonly involved ( 70%), whereas internal iliac artery is involved in 25%. Iliac artery aneurysms are bilateral in approximately 30% cases. External iliac artery is very rare [65]. Those that are smaller than 3.0 cm in diameter tend to be asymptomatic, rarely rupture, and expand slowly; those that are larger than 3.0 cm but smaller than 3.5 cm should be monitored with ultrasonography at 6-month intervals. Iliac artery aneurysms larger than 3.5 cm have a greater tendency to rupture and should be treated expeditiously [66].

In acute, Doppler ultrasonography allows a good detection of the iliac arteries in normal weight patients; however, at present, CT angiography is the most accurate technique to identify and accurately depicts the extravasation of contrast medium in case of aneurysm rupture.

## Venous injury

### Traumatic vascular emergencies

Venous injuries are usually present in the setting of penetrating trauma; however, they are an uncommon imaging finding, likely because of the comorbid trauma-related injuries that either result in death or cause hemodynamic instability requiring immediate surgical intervention before imaging [67].

Modern multidetector CT scanners are able to provide superior image detail of venous trauma.

However, venous opacification, even during the portal venous phase, is often less than that of the arterial structures during the arterial phase because of hemodilution of the contrast material and its elimination by the kidneys. This lessened venous opacification may create inherent and unavoidable limits in the evaluation of the venous structures. Therefore, it is imperative for the radiologist to understand that although current imaging protocols are not designed specifically to evaluate the venous system, venous injuries may nevertheless be identified, with the potential for important alteration of management [68].

Venous injuries can be identified at CT by finding either direct or indirect signs of the injury. Direct signs of vascular injury are diagnostic and include thrombosis and/or occlusion, avulsion and/or complete tear, rupture and active extravasation. Indirect signs of venous injury, such as perivascular hematoma, fat stranding, and vessel wall irregularity, are indeterminate findings because a venous injury may or may not be present. These indirect signs can often be seen in association with other adjacent injuries.

**Figure 8:**
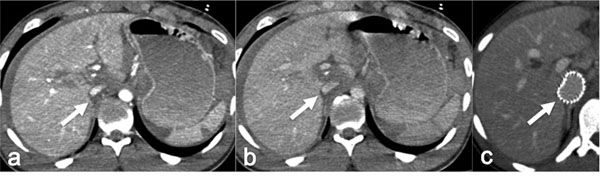
IVC traumatic injury (a) Arterial phase axial CT image depicts a hematoma (arrow) around the IVC close to sovrahepatic veins origin; coexist multiple hepatic and splenic parencymal lacerations*.* (b) Venous phase axial CT image from the same study confirm the IVC hematoma (arrow). (c) Follow-up axial CT image show the precence of endovascular stent in IVC with reestablished caliber (arrow).

The Inferior Vena Cava (IVC) injury are associated with high morbidity and mortality rates. Investigators have reported that more than one-third of patients with an IVC injury die before reaching the hospital, and in-hospital mortality is greater than 60% [69]. Given the high mortality and the common occurrence of other severe comorbid injuries, IVC injuries are not commonly diagnosed at imaging. Imaging options include conventional venography and CT venography [Figure 8]. Few to no data exist to compare conventional venography to CT venography for the evaluation of the IVC in the setting of trauma, therefore each case should be considered individually on the basis of the patient’s clinical presentation.

A common imaging pitfall is mixing of unenhanced blood with contrast material, which can simulate a thrombosis or vessel injury [68].

### Non-traumatic venous emergencies

Risk factors for acute venous occlusion range from prolonged immobilization to hypercoagulability syndromes, trauma, and malignancy. Acute occlusion of the pelvic veins and the inferior vena cava, often due to extension from the femoropopliteal system, represents a major risk for pulmonary embolism.

Acute pelvic vein and inferior vena cava occlusion Isolated pelvic vein occlusion is uncommon in an emergency situation and has been reported mainly as a complication in the postpartum period. Furthermore, thrombosis of the pelvic veins, including the internal iliac veins, can be seen in women with pelvic inflammatory disease and in men with involvement of the prostatic plexus; more frequently, however, acute occlusion of the pelvic veins and the inferior vena cava (IVC) are due to extension from the femoro-popliteal system.

Pelvic vein occlusion should be suspected in patients with abdominal pain, a unilateral pelvic mass, uterine infection and fever that fails to respond to appropriate treatment [70].

The utilization of US has a great importance for the diagnosis of venous occlusion. In the presence of thrombus, the vein is distended and incompressible, sharp definition of the venous wall is lost and the presence of echogenic material inside the lumen may be observed. Very fresh thrombus may be nearly anechoic and consequently not openly perceptible [71]. Color flow is helpful in these cases as the thrombus will appear as a color flow void. The most useful criterion for acute venous occlusion is failure of the vascular lumen to collapse entirely on gentle pressure. In the presence of occlusive thrombus, no flow is detected. Venous flow is generally phasic, decreasing in inspiration and increasing in expiration. A proximal obstruction will prevent such respiratory variation resulting in a continuous flow pattern and will also prevent venous distension normally seen when performing the Valsalva maneuver.

Color flow Doppler imaging is necessary for the evaluation of pelvic vessels but is often limited due to the intrinsic difficulty in plainly outlining the pelvic structures; US imaging in general is also strictly dependent on the technical expertise of the investigator and restricted by patient’s obesity and bowel gas [71].

Both CT scanning and MR imaging can accurately provide the diagnosis of pelvic vein thrombosis. CT venography requires the application of IV contrast agent and is thus contraindicated in patients with renal failure. Pregnancy is another exclusion criterion for CT imaging; however, at present, Contrast-enhanced CT remains the examination of choice for acute pelvic vein and inferior vena cava occlusion .

Venography has long been considered the gold standard for identifying proximal venous occlusion because it allows a complete work-up of the lower limb up to the IVC. It is generally a safe procedure and is often indispensable in case of failure of sonography and in the absence of CT or MR facilities [70].

Acute mesenteric vein occlusion Initially illustrated by Warren and Eberhard in 1935, acute mesenteric vein occlusion is an exceptional and distinctive type of intestinal ischemia. Often idiopathic, precipitating factors could be liver cirrhosis, portal hypertension, neoplasm, intra-abdominal inflammatory diseases, trauma, and hypercoagulable states. Symptoms can be varied and include poorly localized abdominal pain, a change in bowel habits, nausea, vomiting, melena, bloody diarrhea, and ultimately circulatory collapse [72].

As mortality rates for acute mesenteric vein occlusion can be as high as 80%, timely diagnosis is crucial [73].

US and CT imaging are both possible imaging tools in the detection of acute venous occlusion. Contrast-enhanced CT has a sensitivity of 90% and is currently considered the examination of choice [74]; in fact, US modality is afflicted by technical difficulties often due to bowel gas or obesity. With CT, the extent of the occlusion and the collateral venous flow can be appraised.

## Conclusion

Currently, with widely available MDCT technology, the first-line assessment of vascular injury in trauma patients is CTA, instead ultrasound is more used in the non traumatic vascular injuries, but CT is still preferable because the ultrasound has limitations as abdominal distension and obesity, which often do not allow to make the diagnosis. The CTA affords a rapid, accurate, non invasive method of detecting vascular injury and appropriately triaging patients to receive the requisite intervention, when necessary.

## Competing interests

The authors have no conflict of interest.

## Declarations

This article has been published as part of *Critical Ultrasound Journal* Volume 5 Supplement 1, 2013: Topics in emergency abdominal ultrasonography. The full contents of the supplement are available online at http://www.criticalultrasoundjournal.com/supplements/5/S1. Publication of this supplement has been funded by the University of Molise, University of Siena, University of Cagliari, University of Ferrara and University of Turin.
